# Achenbach’s Syndrome: The Case of a Benign Paroxysmal Blue Finger

**DOI:** 10.7759/cureus.103668

**Published:** 2026-02-15

**Authors:** Alina Stoica, Jamal Khudr, Kirti Garude, Aftab Siddiqui

**Affiliations:** 1 Plastic and Reconstructive Surgery, Countess of Chester Hospital, Chester, GBR; 2 Plastic Surgery, Manchester University Foundation Trust, Manchester, GBR

**Keywords:** achenbach’s syndrome, blue finger syndrome, finger hematoma, paroxysmal finger hematoma, spontaneous digital hematoma

## Abstract

Achenbach's syndrome, or paroxysmal finger haematoma, is a rare but benign vascular phenomenon characterized by the sudden onset of pain, swelling, and dramatic blue-purple discoloration of a finger, typically without trauma or systemic illness. Although benign and self-limiting, it is frequently under-recognized and often triggers emergency referrals due to its dramatic clinical appearance. We present a case of a 45-year-old man who developed acute volar finger ecchymosis while demonstrating cardio-pulmonary resuscitation compressions, with no underlying pathology identified. A detailed literature review highlights the condition's typical presentation, diagnostic challenges, and potential for misdiagnosis as more serious vascular events, such as digital ischaemia or vasculitis. Our case emphasizes the critical role of clinical recognition in avoiding unnecessary investigations and treatments. Increased awareness of Achenbach's syndrome among clinicians is essential to ensure accurate diagnosis, patient reassurance, and optimal use of healthcare resources.

## Introduction

Achenbach's syndrome is a rare benign condition characterised by sudden and unexplained bruising of the fingers. It is also known as 'acute idiopathic blue finger' and as paroxysmal finger haematoma. Initially described by Walter Achenbach, a German physician, he documented in 1958 a series of patients who presented with ‘finger apoplexy’ or recurrent spontaneous haematoma of the hand [1]. This condition continues to be under-recognised, as it has primarily been reported throughout the years in case reports and small case series. This entity has been described using multiple terms in the literature, including paroxysmal hand haematoma, finger apoplexy, painful blue finger, and ultimately Achenbach’s syndrome [1,2]. With no consistent systemic pathology or precipitating factors identified, the exact cause of Achenbach’s syndrome remains unknown.

The perception of extreme rarity comes from the limited number of Achenbach's syndrome presentations reported within the literature [2]. Mild cases, which go under-reported, may have significantly contributed to this observation, as a cross-sectional study that involved 802 members of the population in France found a prevalence of 12.4% among women and 1.2% among men [3]. With a higher affliction rate in women, this syndrome is also more likely to affect middle-aged adults. Most patients tend to be women in their fourth to sixth decade of life; it can also affect men and older and younger individuals, albeit less frequently. The phenomenon is sporadic, although there are reports of occurrences within families that may indicate a possible genetic predisposition. There is no similar association with race or ethnicity, with reports describing the condition affecting both Caucasian and Asian patients available in the literature [2].

Our case reflects a classical presentation of Achenbach's syndrome, mirroring the characteristic features described in the literature: sudden onset of pain, swelling, and volar finger ecchymosis with sparing of the fingertip and nail bed.

## Case presentation

We present the case of a middle-aged patient, a 45-year-old right-handed man who works as a resuscitation training instructor. He presented to our accident and emergency (A&E) department with sudden-onset pain (same day of presentation) and swelling affecting his right middle finger. He denied any history of trauma prior to the evolution of symptoms. He reported that whilst demonstrating chest compressions as part of a cardio-pulmonary resuscitation technique earlier in the day, he felt a sharp pain localised to the base of his right middle finger. Over the next hour, he noticed an alarming bluish discoloration to his finger as it started swelling over the middle phalanx. This dramatic change in colour caused significant distress regarding potential vascular compromise to the finger. He had no significant past medical history, no known coagulopathies, no peripheral vascular disease, and was on no regular medications or anti-coagulants. He was generally in good health and did not smoke.

**Figure 1:**
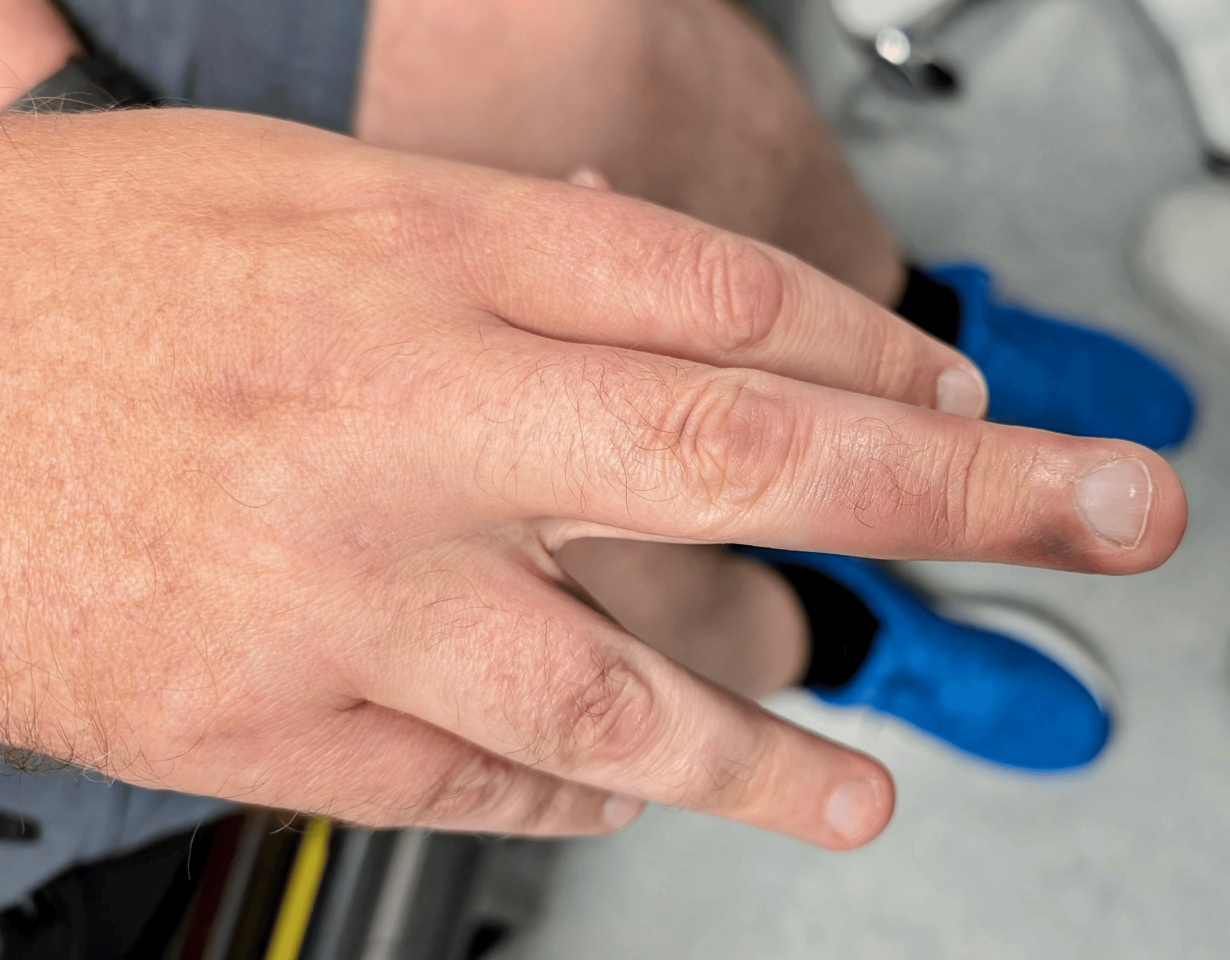
Right middle finger discolouration with nail bed spared The figure depicts the right middle finger showing bluish-purple discolouration, with sparing of the nail bed.

**Figure 2:**
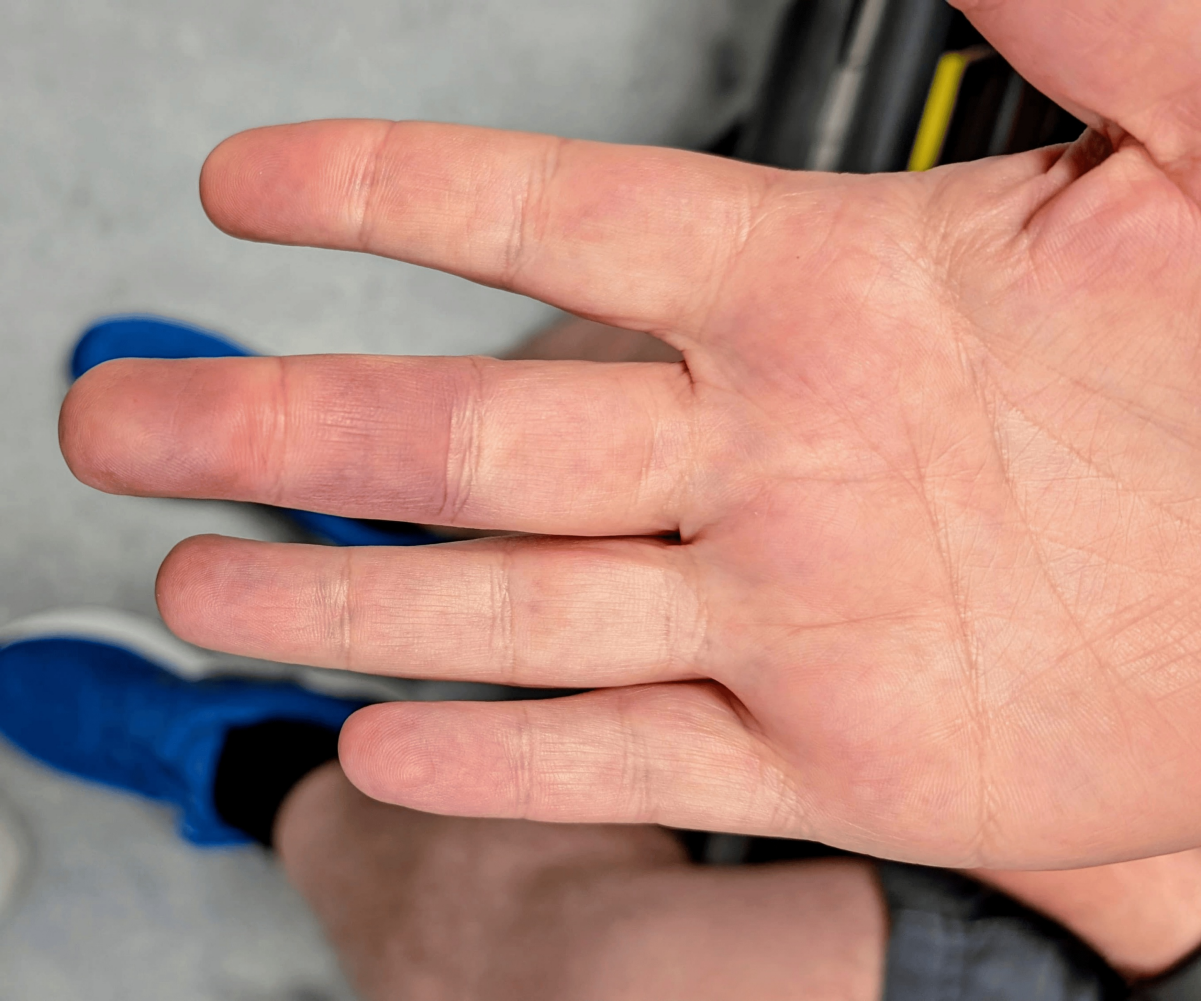
Right middle finger discolouration palmar aspect The figure depicts the palmar aspect of the right middle finger showing bluish-purple discolouration, which is well defined primarily located on the palmar aspect of the finger, with sparing of the nail bed.

On general examination of the patient, he was anxious but not in visible distress from the finger pain. His vital signs were normal. Closer inspection of the right hand identified isolated bluish-purple discolouration to the volar aspect of the right middle finger, extending from the proximal interphalangeal (PIP) joint to the middle phalanx. This bruising was well demarcated and associated with moderate swelling of the finger (Figures 1-2). The nail bed was unaffected, and the fingertip appeared well-perfused and pink with no pallor or cyanosis. The finger was warm, with a capillary refill time at the distal pulp of under two seconds, in keeping with the non-affected digits on both the ipsilateral and contralateral hand. On palpation, mild tenderness was noted over the bruised area; however, this had significantly reduced by the time of examination, which was undertaken approximately four hours after onset. Full range of motion was noted in the finger, with limitation on full flexion due to the underlying swelling. No signs of trauma were visible, including a wound, puncture, or infection. No other abnormality was noted on examining all other fingers and the other hand. Both radial and ulnar pulses were present and equal at the wrist. The rest of the physical examination was unremarkable. When placed in an oxygen saturation probe, the monitor indicated a 100% saturation level in the finger, indicating an intact arterial circulation. 

**Figure 3:**
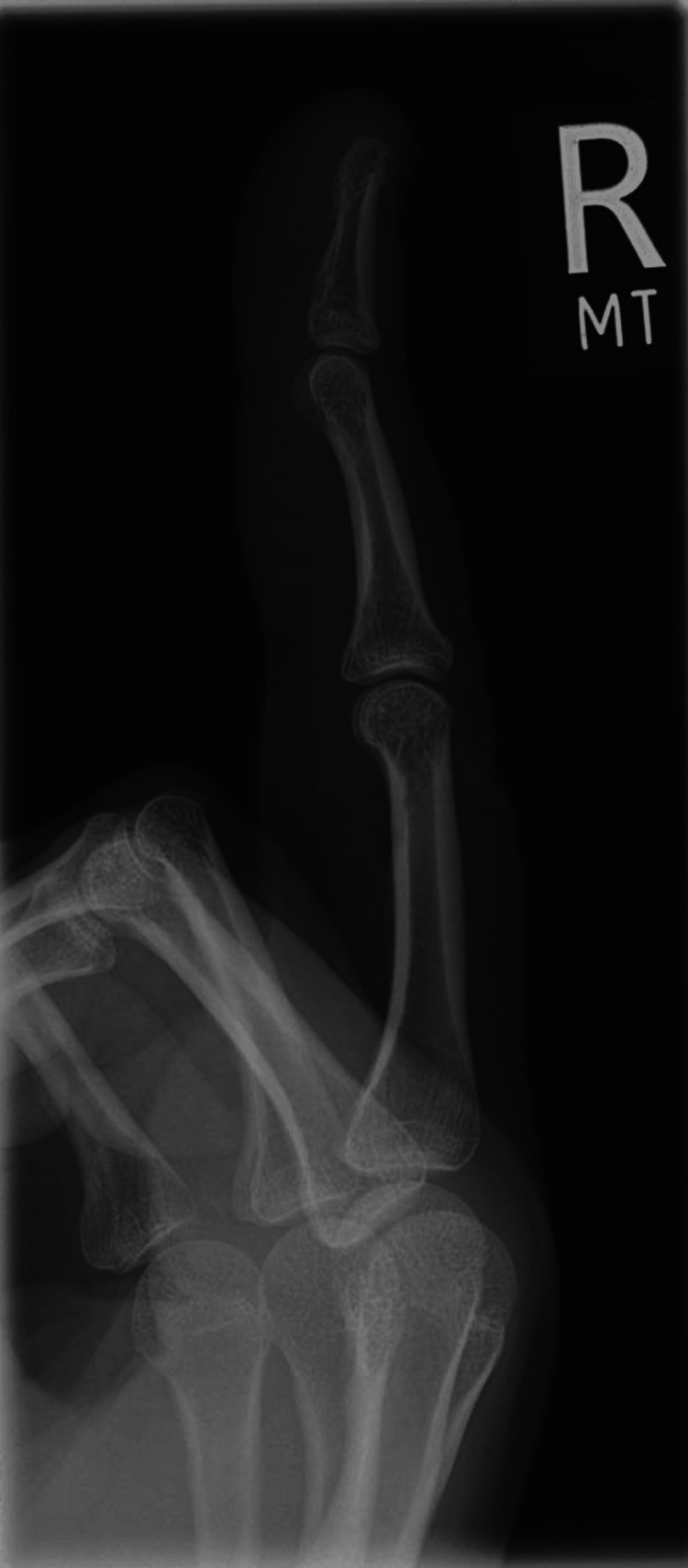
XR lateral view right middle finger

**Figure 4:**
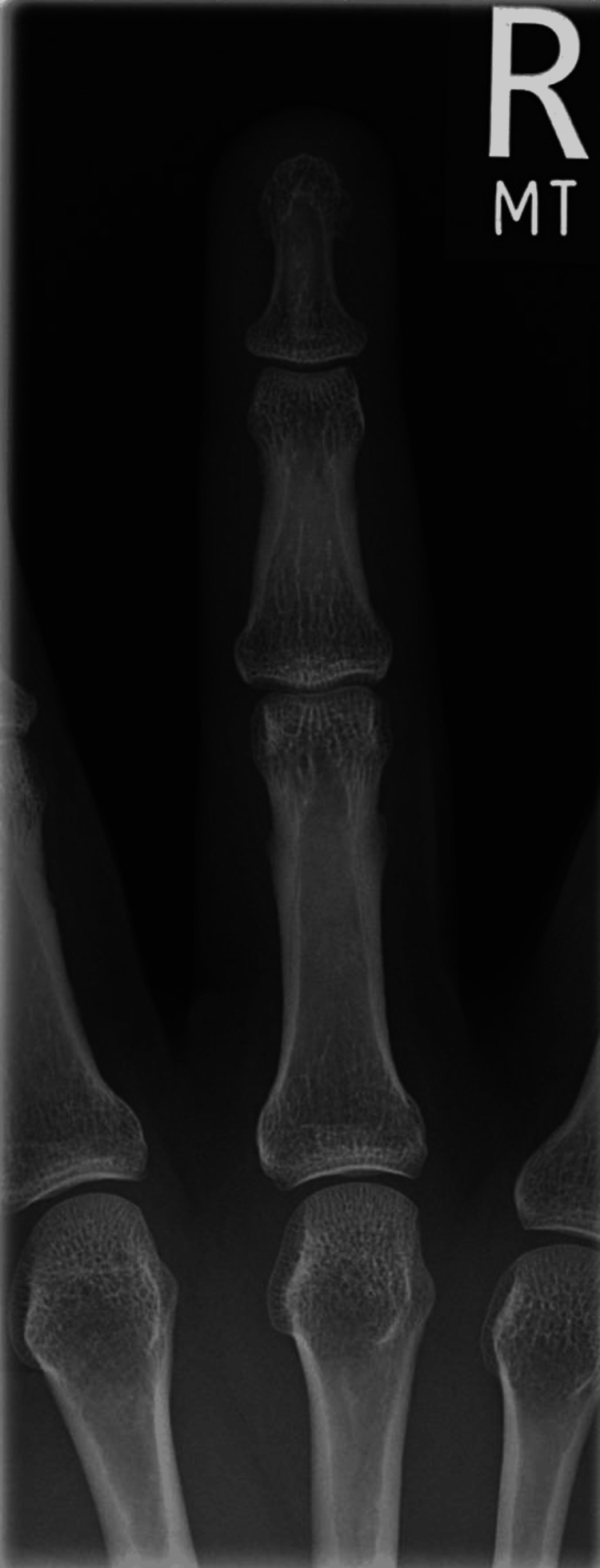
XR antero-posterior view right middle finger

Investigations were performed in the first instance to rule out other potential causes. A plain film radiograph of the affected hand revealed no underlying fractures or bony abnormalities (Figures 3-4). Laboratory tests were all within normal limits. A coagulopathy was unlikely, considering normal platelet levels and the absence of prolonged bleeding times. Inflammatory markers, including C-reactive protein and erythrocyte sedimentation rate, were also normal and highlighted the absence of an underlying autoimmune or inflammatory pathology. 

We considered possibilities of Raynaud’s phenomenon or vasculitis processes; however, the intact circulation with normal results was reassuring. We requested an ultrasound Doppler study to assess blood flow in the digit. However, when the patient was contacted to attend for his appointment a week after initial presentation, he did not attend, citing resolution of his symptoms. 

At this stage, with serious underlying pathology excluded, we considered a diagnosis of Achenbach’s syndrome. This was a pathology that we had not personally seen before, and after review of the literature, we recognised the classic presentation of this syndrome. With this conclusion at hand, invasive interventions and further surgical management were avoided.

The patient was managed conservatively, with initial monitoring in the emergency department for several hours to ensure no sudden compromise to finger vascularity. Oral non-opioid analgesics in the form of paracetamol were given for pain and provided adequate relief. At the time of discharge from our A&E, approximately six hours after onset, the patient reported the pain at a minimal level with no further progression in the extent of the discolouration. We provided strict safety netting advice to present if there was any further deterioration in symptoms and arranged for an outpatient clinic appointment in two weeks.

The patient was then re-evaluated in our hand surgery clinic and reported spontaneous resolution over the intervening days. On examination in the clinic, the swelling and discolouration had completely resolved with no signs of residual discolouration nor recurrence. He had resumed his normal daily activity without any limitation and had no recurrence in the interim. Given the benign course, we explained the presumptive diagnosis of Achenbach’s syndrome to the patient and counselled him that the condition is benign and, should it recur, it would resolve spontaneously once more. In case of recurrence, we advised him to present for further evaluation to ensure the absence of other causative pathologies. The diagnosis was communicated to the patient’s primary care physician to ensure continuity of care and awareness of this condition.

**Figure 5:**
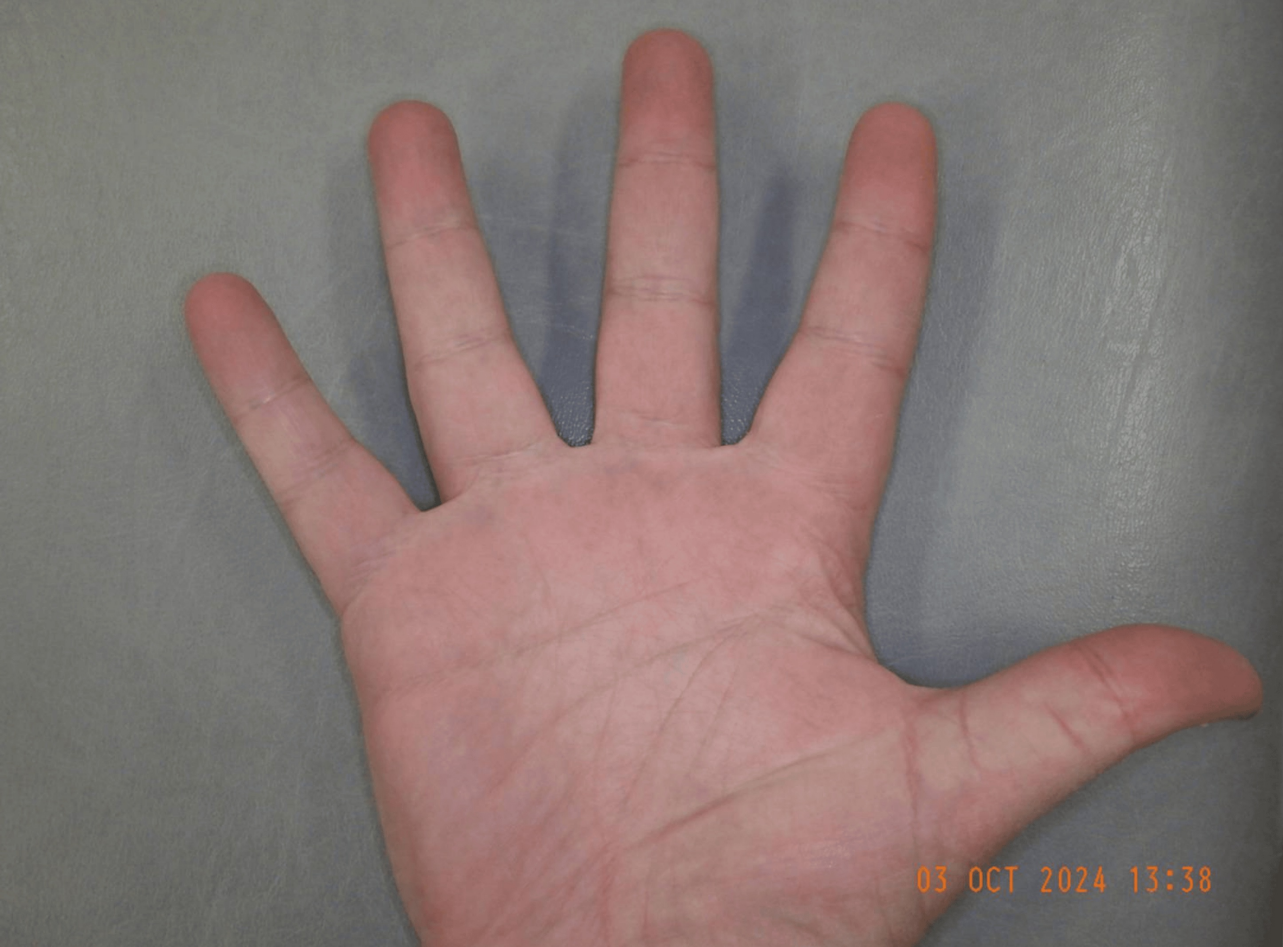
Right middle finger palmar aspect showing normal colour on follow-up

**Figure 6:**
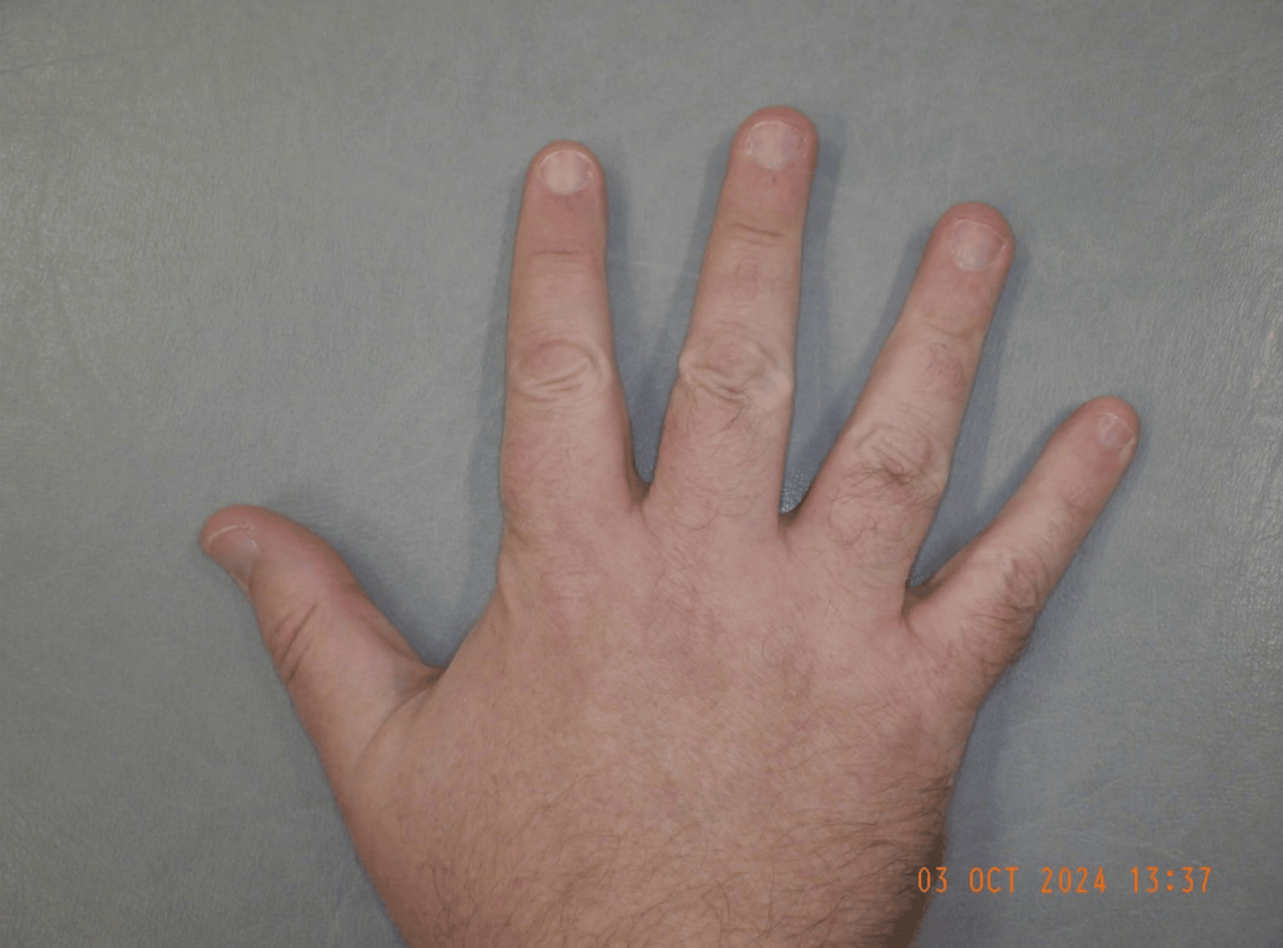
Right middle finger dorsum showing normal colour on follow-up

Figures 5-6 depict the patient’s right middle finger on follow-up two weeks after the episode, with bruising completely resolved and normal appearance restored.

## Discussion

Achenbach's syndrome, although rare and benign, poses a significant diagnostic challenge due to its dramatic presentation and limited awareness among clinicians. With no consistent systemic pathology or precipitating factors identified, the exact cause of Achenbach’s syndrome remains unknown. It is important to note that there is no association with any known vascular abnormalities, including peripheral arterial disease, clotting disorders, and vasculitides. This is corroborated in reported cases, as patients typically do not have any predisposing coagulopathy, and clotting profiles are more commonly normal on blood results. The current theory suspects spontaneous subcutaneous haemorrhage secondary to increased fragility within the capillaries and venules, i.e., the small blood vessels, within the fingers [1]. In most cases, the condition is idiopathic with no clear trigger; however, in about one-third of cases, patients may report some strain or trivial minor trauma to the hand before the onset of symptoms, such as doing chores around the house or other repetitive movements. At present, this combination of capillary fragility with a superimposed shearing force is thought to lead to a self-limiting bleed within the finger pulp and volar soft tissue. As such, this spares the nail bed of any discolouration while following a benign course with no compromise of arterial blood flow and distal circulation [4].

Achenbach syndrome typically presents quite dramatically, yet with classical characteristics. These usually include an acute onset of pain that patients often describe as stinging, burning, or even a sudden 'pinch' that is limited to one finger with no clear precipitating cause. The affected finger then becomes increasingly swollen as it develops a spontaneous and striking blue-purple discolouration, or ecchymosis, of the underlying skin within minutes to hours. The index and middle fingers are most likely to be affected, followed by the ring finger. Discolouration is usually first noted at the volar aspect of the finger, particularly at the palmar digital crease or middle phalanx. The sparing of the fingertip, including the nail bed and distal phalanx, is a hallmark of Achenbach syndrome that helps in distinguishing it from acute ischaemic events, which would typically affect the entire finger [5]. Some patients may describe the finger 'numb' or 'tingly' with a sensation of pressure, tightness, or itching [6].

On examination, the discolouration and bruising are well-demarcated and limited to the volar finger and are associated with swelling and tenderness over the involved area. Circulation to the finger remains intact, despite the concerning appearance of the finger, and this can be confirmed on palpation of a warm finger, with a normal capillary refill. Motor examination is normal with potential limitation due to pain and swelling of the digit. Symptoms are localised to the finger, with no systemic stigmata visible on holistic examination of the patient [7].

The differential diagnosis for paroxysmal finger haematoma is broad, encompassing several vascular, rheumatological, and haematological conditions that must be distinguished from this benign presentation. For instance, Raynaud’s phenomenon is commonly seen with cold-induced reversible triphasic colour changes in distal digits, following sequelae of pallor, then cyanosis, and finally redness. In contrast to Achenbach's, it typically affects the fingertips. Acute limb ischaemia or arterial embolus, also known as ‘blue finger syndrome’, typically presents with absent pulses, cool skin on palpation, whole digit involvement, with significant pain out of proportion due to underlying ischaemia, which would not be present in Achenbach's [8]. Vasculitis or connective tissue disorders or digital vasculitis secondary to an underlying rheumatological origin, such as systemic lupus erythematosus or rheumatoid arthritis, can cause infarcts or haemorrhagic lesions. These are usually a stigma of a larger clinical picture, including multisystem involvement that corresponds with abnormal inflammatory and autoimmune markers that would otherwise be normal in Achenbach’s [9]. Microemboli, either atherosclerotic or cardiac in origin, are rare in nature and increasingly prevalent with age. Patients present with ischaemic pain in the finger with signs of arterial disease, which are absent in Achenbach's. Gardner-Diamond syndrome (psychogenic purpura) is a rare syndrome where mostly young women present with painful bruising and psychological stress. This can present on extremities and trunk; however, they tend to be multiple and not limited to the hands. This presentation, in combination with the associated psychological factors, helps differentiate it from Achenbach’s. Furthermore, regarding haematologic causes of bruising, spontaneous bruising can be the cause of thrombocytopenia or other coagulopathies; however, these abnormalities would be reflected on laboratory testing, with bruising affecting multiple areas. Clinically, distinguishing Achenbach’s syndrome from conditions such as Raynaud’s phenomenon, digital artery embolism, or vasculitis disorders is essential. Unlike ischaemic conditions, the affected finger is warm, perfused, and has normal capillary refill, with symptoms resolving by themselves. This marked difference shows how important it is to use clinical judgment [5].

Achenbach’s syndrome is a clinical diagnosis based on history and examination. However, a workup may still be used to help rule out other, more serious causes for the presentation. In most cases, laboratory testing yields normal results when examining blood count, coagulation studies, inflammatory markers, and autoimmune serologies. These all help rule out bleeding diathesis, inflammatory processes, or autoimmune syndromes [2].

Imaging is typically undertaken due to the dramatic appearance of the finger. Plain radiographs would most likely be normal or only show a slight soft tissue swelling while ruling out any underlying bony injury or osteolytic pathology. Vascular imaging may be performed when there is suspicion of ischaemia to better assess blood flow. Doppler ultrasound or arterial duplex would usually yield patent arteries. Angiography is an invasive procedure that may further confirm patency of the arteries or indicate a transient non-specific vasospasm [10]. The use of capillaroscopy in one study has exhibited microhaemorrhages in the affected finger, which would further reinforce the suspicion of an underlying superficial vascular leakage as the causative pathology. If testing is unremarkable and clinical findings align with a diagnosis of Achenbach’s, exhaustive testing is not required, as this will only yield negative or incidental results [11].

Achenbach’s syndrome carries an excellent prognosis as a benign condition. Most patients experience a spontaneous resolution of discolouration and pain within a few days, typically three to seven days and up to two weeks, with no residual symptoms. The colour of the bruising surprisingly fades away without experiencing the multicoloured evolution of green, yellow, and brown, typically present with traumatic bruising. It is important to reassure patients that there are no lasting sequelae with this condition: no tissue necrosis, no chronic pain, and most importantly, no loss of function or loss of the finger as it returns to normal [7].

Patients are most likely to experience recurrence of the syndrome, whether in the same digit or a different one. The condition remains benign and self-limiting with no long-term consequences even with recurrent bouts. With recurrence, it is important to occasionally confirm no other cause has potentially developed, but otherwise, the mainstay of management remains conservative with appropriate recognition, patient education, and reassurance [12].

Early recognition of Achenbach's syndrome is critical in guiding appropriate management and reducing unwarranted diagnostic workup. In our case, the diagnosis was ultimately clinical, supported by normal radiographic and laboratory investigations, and a self-limiting course. This aligns with findings from previous reports, including those by Kordzadeh et al. (2016), which highlight that misdiagnosis may lead to over-treatment [13]. For instance, Chen et al. (2024) described a case where a patient was escalated to tertiary vascular services and unnecessarily initiated on anticoagulation therapy for presumed digital ischaemia. Our patient similarly underwent initial imaging and blood work, which may have been avoided with earlier identification of the syndrome’s pathognomonic features [14].

Importantly, the benign nature of the syndrome should not downplay the psychological burden it places on patients. The abrupt visual transformation of the finger can be alarming, often prompting emergency consultations. In our case, the patient presented to A&E in considerable distress. Here, patient education and physician reassurance play a pivotal role. Increasing awareness among primary and emergency care providers can streamline patient journeys and reduce healthcare costs. Moreover, awareness of potential recurrence is vital, and patients should be reassured about its non-progressive nature.

This case reinforces the need for heightened clinical awareness. Achenbach's syndrome, though visually striking, follows a benign trajectory. When recognised promptly, it spares the patient from follow-up investigations and interventions meant for more serious vascular conditions.

## Conclusions

Achenbach's syndrome is an under-recognized but benign condition characterised by the sudden onset of finger bruising. This case highlights the importance of clinician awareness and pattern recognition, which are important for differentiating it from more serious vascular or autoimmune conditions. A clinical diagnosis based on history and physical findings should prompt reassurance and conservative management, avoiding unnecessary tests or invasive procedures.

Timely identification not only prevents iatrogenic harm but also alleviates patient anxiety. While the condition is self-limiting, patient follow-up remains essential to confirm resolution and monitor for recurrence. Ultimately, improving clinician familiarity with Achenbach’s syndrome supports better patient outcomes and a more efficient use of healthcare resources.
